# Analysis of Damage and Permeability Evolution for Mudstone Material under Coupled Stress-Seepage

**DOI:** 10.3390/ma13173755

**Published:** 2020-08-25

**Authors:** Bin Liu, Jinlan Li, Quansheng Liu, Xuewei Liu

**Affiliations:** 1 State Key Laboratory of Geomechanics and Geotechnical Engineering, Institute of Rock and Soil Mechanics, Chinese Academy of Sciences, Wuhan 430071, China; liubin@whrsm.ac.cn; 2 School of Civil Engineering and Environment, Hubei University of Technology, Wuhan 430068, China; 3 The Key Laboratory of Safety for Geotechnical and Structural Engineering of Hubei Province, School of Civil Engineering, Wuhan University, Wuhan 430072, China; clycir@163.com

**Keywords:** mudstone material, damage model, permeability evolution, finite element method, coupled stress-seepage

## Abstract

Mudstone material in a deep roadway is under the coupled stress-seepage condition. To investigate the permeability change and damage development during rock excavation in roadways, a stress-seepage damage coupling model has been proposed. In this model, damage capacity expansion of mudstone material is considered as the initiation and propagation of micro-cracks and the fracture penetration. A damage variable is introduced into the proposed model based on the principle of minimum energy consumption. As a result, an elastoplastic damage constitutive equation is established. Then, the permeability evolution equation describing the micro-macro hydraulic behavior of mudstone is deduced via percolation theory, which can describe the characteristics of sudden permeability change after rock capacity expansion. Furthermore, a finite element model is established based on commercial finite element software-ABAQUS. The numerical model was firstly verified by comparison between experimental and simulation results. On the basis of it, numerical investigation of the temporal and spatial evolution law of pore pressure, damage and permeability coefficient during roadway excavation is undertaken. The numerical results indicate that with increase of construction time, pore pressure first increases and then decreases, while the damage zone and permeability coefficient increase gradually and finally nearly keep constant. The proposed coupling model and finite element method can describe damage and permeability evolution for mudstone material under coupled stress-seepage well.

## 1. Introduction

In deep resource exploitation, geothermal resource development, deep tunnel excavation, underground storage of nuclear waste, etc., multi-field coupling problems are involved. Before underground construction, the rock mass has low permeability, and the seepage-stress coupling effect is not obvious. The rock mass stress redistribution caused by the excavation of a tunnel (roadway) leads to damage of the surrounding rock. As the fracture expands, the adjustment and migration of the stress field, seepage field and damage evolution process as well as the interaction among them are very significant, seriously affecting surrounding rock stability in an underground rock project [1,2,3,4]. Therefore, it is of great theoretical significance and engineering application value for stability control and safety evaluation of surrounding rock in underground engineering to study rock seepage-stress-damage coupling, understand the interaction mechanism of rock mass damage and seepage, and predict the possible damage mode and range of the rock mass [5,6].

Many rich research results have been obtained in the study of multi-field coupling of rock mass. Initial studies did not consider the impact of damage. Some scholars combined geomechanics with seepage mechanics to study the coupling of porous media and fractured rock mass. Several seepage-stress coupling models of rock mass were established [7,8,9,10]. With the deepening of research on the coupling of rock masses, it was found that mechanical properties and hydraulic characteristics of rock mass change obviously after damage and rupture. The effect of damage needs to be considered on coupling of seepage-stress damage. Kelsall [11] analyzed the effect of underground construction on the surrounding rock, and studied the variation law of surrounding rock damage caused by excavation and its permeability evolution. Yang [12] proposed a seepage-damage coupling analysis model for the seepage of fractured rock mass to discuss the effect of seepage on the mechanical behavior as well as the effect of stress state on fracture permeability. Zhang [13] established the seepage-stress coupling model considering damage according to the principle of the same principle axis of strain, damage and additional permeability. Ran [14] introduced the concept of mutual coupling of plastic damage evolution and seepage into the Mohr–Coulomb failure criterion, gave the permeability evolution and damage evolution equations of mudstone, and analyzed stability of mudstone borehole wall using a seepage-stress damage coupling model. 

In the current theoretical analysis of seepage-stress damage coupling [15,16,17,18,19,20,21,22,23], the permeability evolution model cannot describe the characteristics of sudden permeability change after rock capacity expansion. According to the permeability evolution characteristics of mudstone, the sudden change of permeability evolution before and after capacity expansion in the process of total stress-strain is analyzed. Furthermore, the permeability evolution equation describing the micro-macro hydraulic behavior of mudstone is deduced based on percolation theory. Finally, the seepage-stress damage coupling model of mudstone is established. Furthermore, the construction process of a roadway is simulated to validate the effectiveness of the proposed theoretical model using numerical simulation.

## 2. Stress-Seepage Damage Coupling Model

In this section, the mechanical behavior characteristics of mudstone were first analyzed and then the mudstone excavation damage mechanism was revealed. With the help of the principle of minimum energy consumption, the damage evolution equation describing mudstone macro mesoscopic mechanical behavior and failure characteristics and an elastoplastic damage constitutive model was established. Combined with the stress balance equation and continuous equations, the stress-seepage damage coupling model was finally proposed. 

### 2.1. Elastoplastic Damage Constitutive Equation

When describing the plastic behavior of mudstone, it is assumed that it is subject to the Coulomb yield criterion, and then the elastoplastic constitutive relation in incremental form is:

(1) $${\left[C\right]}_{p}=\frac{\left[C^{*}\right]\left(\frac{\partial g^{p}}{\partial \sigma^{\prime }}\right){\left(\frac{\partial f}{\partial \sigma^{\prime }}\right)}^{T}\left[C^{*}\right]}{A+{\left(\frac{\partial f}{\partial \sigma^{\prime }}\right)}^{T}\left[C^{*}\right]\left(\frac{\partial g^{p}}{\partial \sigma^{\prime }}\right)}$$

In Equation (1), $\left[C^{*}\right]=\left(1-D\right){\left[C\right]}_{e}$. where parameter D is the damage variable, parameter ${\left[C\right]}_{e}$ is the elastic matrix, parameter ${\left[C\right]}_{p}$ is the plastic matrix, parameter f is the yield function, parameter $g^{p}$ is the plastic potential function, and parameter A the modulus characterizing hardening or softening. 

Because of the tensile stress concentration, the micro-fractures in the mudstone initiation, propagation and connection, will finally cause the non-linear deformation of mudstone at the damage capacity expansion stage. Based on the principle of minimum energy consumption, the damage variable *D* is considered as an internal variable, and the nonlinear principal strain $\varepsilon_{i}^{N}\left(i=1,2,3\right)$ of mudstone caused by loading is set as the energy dissipation mechanism of mudstone in the damage process. According to the principle of minimum energy dissipation, the rate of energy dissipation ϕ(t) can be expressed as:

(2) $$\phi \left(t\right)=\sigma_{i}{\dot{\varepsilon }}_{i}^{N}\left(t\right)$$

where, $\sigma_{i}\left(i=1,2,3\right)$ is the nominal principal stress; ${\dot{\varepsilon }}_{i}^{N}\left(t\right)$ is the rate of nonlinear principal strain at time t; t is the time parameter of energy dissipation process.

For isotropic damage, the stress-strain relationship at any time t can be expressed as:

(3) $$\left\{ \begin{array}{l} \varepsilon_{1}\left(t\right)=\frac{1}{\left[1-D\left(t\right)\right]E}\left[\sigma_{1}-\mu \left(\sigma_{2}+\sigma_{3}\right)\right]\\ \varepsilon_{2}\left(t\right)=\frac{1}{\left[1-D\left(t\right)\right]E}\left[\sigma_{2}-\mu \left(\sigma_{1}+\sigma_{3}\right)\right]\\ \varepsilon_{3}\left(t\right)=\frac{1}{\left[1-D\left(t\right)\right]E}\left[\sigma_{3}-\mu \left(\sigma_{2}+\sigma_{1}\right)\right] \end{array}\right.$$

The following can be obtained from the Equation (3):

(4) $$\left\{ \begin{array}{l} {\dot{\varepsilon }}_{1}^{N}\left(t\right)=-\frac{\dot{D}\left(t\right)}{{\left[1-D\left(t\right)\right]}^{2}E}\left[\sigma_{1}-\mu \left(\sigma_{2}+\sigma_{3}\right)\right]\\ {\dot{\varepsilon }}_{2}^{N}\left(t\right)=-\frac{\dot{D}\left(t\right)}{{\left[1-D\left(t\right)\right]}^{2}E}\left[\sigma_{2}-\mu \left(\sigma_{1}+\sigma_{3}\right)\right]\\ {\dot{\varepsilon }}_{3}^{N}\left(t\right)=-\frac{\dot{D}\left(t\right)}{{\left[1-D\left(t\right)\right]}^{2}E}\left[\sigma_{3}-\mu \left(\sigma_{2}+\sigma_{1}\right)\right] \end{array}\right.$$

Introduce Equation (4) into the Equation (2) to obtain:

(5) $$\phi \left(t\right)=-\frac{\dot{D}\left(t\right)}{{\left[1-D\left(t\right)\right]}^{2}E}\left[\begin{array}{l} \sigma_{1}^{2}+\sigma_{2}^{2}+\sigma_{3}^{2}-2\mu (\\ \sigma_{1}\sigma_{2}+\sigma_{2}\sigma_{3}+\sigma_{3}\sigma_{1}) \end{array}\right]$$

The constraint condition of mudstone in the process of energy dissipation is (failure criterion of mudstone):

(6) $$\left\{ \begin{array}{l} F_{1}\left(\sigma \right)=\sigma_{1}-m^{\prime }\sigma_{3}-n^{\prime }=0\\ F_{2}\left(\sigma \right)=\sigma_{3}-\sigma_{t}=0 \end{array}\right.$$

where, $m^{\prime }=\frac{1+\sin\varphi_{0}}{1-\sin\varphi_{0}}$, $n^{\prime }=\frac{2c_{0}\cos\varphi_{0}}{1-\sin\varphi_{0}}$, $c_{0}$ is the initial cohesion, and $\varphi_{0}$ is the initial internal friction angle.

According to the principle of minimum energy dissipation, under the condition of Equation (6), the Lagrange multiplier $\lambda_{1}$ and $\lambda_{2}$ are introduced into the Equation (5), and the Equation (7) can be obtained from the stationary values.

(7) $$\left\{ \begin{array}{l} \frac{\partial \left[\phi \left(t\right)+\lambda_{1}F_{1}\right]}{\partial \sigma_{i}}=0\\ \frac{\partial \left[\phi \left(t\right)+\lambda_{2}F_{2}\right]}{\partial \sigma_{i}}=0 \end{array}\right.$$

After the arrangement, the damage evolution equation is shown in Equation (8).

(8) $$D=1-\exp\left[\frac{\lambda_{1}\left(1-m^{\prime }\right)+\lambda_{2}}{2\varepsilon_{v}}+C\right]$$

where $\varepsilon_{v}$ is the volumetric strain, $\lambda_{1}$, $\lambda_{2}$, and C are the material constants to be determined by experiment.

### 2.2. Stress Balance Equation

The study of seepage-stress in saturated-unsaturated rock and soil medium is two or more fluids flowing in pores. In this work, only water and air are considered in the pores, and the viscosity of water are not considered. besides, the unsaturated zone is connected with the atmosphere, as a result, the pressure at unsaturated zone is equal to the atmospheric pressure.

For saturated-unsaturated geomaterials, the effective stress expression can be written as following:

(9) $$\sigma =\sigma^{\prime }-m\bar{p}$$

where $m={\left[\begin{array}{cccccc} 1, & 1, & 1, & 0, & 0, & 0 \end{array}\right]}^{T}$.

The effective stress-strain constitutive relation in incremental form is:

(10) $$d\sigma^{\prime }=C_{ep}\left(d\varepsilon -d\varepsilon_{l}\right)$$

where, $C_{ep}$ is the elastoplastic matrix; $d\varepsilon_{l}$ is the strain increment due to pore fluid pressure, $\varepsilon_{l}=-m\frac{d\bar{p}}{3K_{S}}$.

According to the virtual work principle, the stress balance equation expressed in incremental form is:

(11) $$\int_{\Omega }\delta \varepsilon^{T}d\sigma d\Omega -\int_{\Omega }\delta u^{T}dbd\Omega -\int_{\Gamma }\delta u^{T}d\hat{t}d\Gamma =0$$

where, δε is the virtual strain; δu is the virtual displacement; b is physical strength; $\hat{t}$ is surface force.

According to Equations (9)–(11) and by time derivation, it can be obtained that:

(12) $$\int_{\Omega }\delta \varepsilon^{T}C_{ep}\left(\frac{d\varepsilon }{dt}+m\frac{1}{3K_{S}}\frac{d\bar{p}}{dt}\right)d\Omega -\int_{\Omega }\delta \varepsilon^{T}m\frac{d\bar{p}}{dt}d\Omega -\int_{\Omega }\delta u^{T}\frac{db}{dt}d\Omega -\int_{\Gamma }\delta u^{T}\frac{d\hat{t}}{dt}d\Gamma =0$$

Assuming that the pore pressure is constant at atmospheric pressure, then the derivative of the average pore fluid pressure over time can be reduced to:

(13) $$\frac{d\bar{p}}{dt}=s_{w}\frac{dp_{w}}{dt}+p_{w}\frac{ds_{w}}{dt}$$

For unsaturated problems, saturation $s_{w}$ is a function of capillary pressure $p_{c}$. When pore pressure is constant at atmospheric pressure, the saturation $s_{w}$ is also a function of pore water pressure. The change in saturation can then be expressed as:

(14) $$\frac{ds_{w}}{dt}=\frac{ds_{w}}{dp_{w}}\frac{dp_{w}}{dt}=\xi \frac{dp_{w}}{dt}$$

where, ξ is determined by the test curve between capillary pressure $p_{c}$ and saturation $s_{w}$.

Substituting Equations (13) and (14) into Equation (12), the balance equation is obtained as in Equation (15).

(15) $$\begin{array}{l} \int_{\Omega }\delta \varepsilon^{T}C_{ep}\frac{d\varepsilon }{dt}d\Omega +\int_{\Omega }\delta \varepsilon^{T}C_{ep}\left(m\frac{\left(s_{w}+p_{w}\xi \right)}{3K_{S}}\frac{dp_{w}}{dt}\right)d\Omega \\ -\int_{\Omega }\delta \varepsilon^{T}m\left(s_{w}+p_{w}\xi \right)\frac{dp_{w}}{dt}d\Omega =\int_{\Omega }\delta u^{T}\frac{db}{dt}d\Omega +\int_{\Gamma }\delta u^{T}\frac{d\hat{t}}{dt}d\Gamma \end{array}$$

### 2.3. Continuous Equation

According to the principle of mass conservation, for a given volume of rock, the amount of water that flows into this volume within time dt should equal the increase in its internal water storage. Fluid seepage is described by Darcy’s law. After derivation, the continuity equation of seepage is shown as Equation (16).

(16) $$\begin{array}{l} s_{w}\left(m^{T}-\frac{m^{T}C_{ep}}{3K_{S}}\right)\frac{d\varepsilon }{dt}-\nabla^{T}\left[K\left(\frac{\nabla p_{w}}{\rho_{w}}-g\right)\right]+\\ \left\{\xi n+n\frac{s_{w}}{K_{w}}+s_{w}\left[\frac{1-n}{3K_{S}}-\frac{m^{T}C_{ep}m}{{\left(3K_{S}\right)}^{2}}\right]\left(s_{w}+p_{w}\xi \right)\right\}\frac{dp_{w}}{dt}=0 \end{array}$$

where, K is the product of permeability coefficient k and water density; $\rho_{w}$ is water density; g is gravity acceleration vector; $K_{w}$ is volume modulus of water; n is porosity.

## 3. Permeability Evolution Model of Mudstone Material

When the mudstone is locally fractured due to tension/shear forces, the permeability coefficient increases drastically. The evolution of permeability with displacement is directly related to strain localization or damage. The permeability evolution model of mudstone is mainly to analyze the relationship between permeability coefficient, stress (strain) and damage. Then, we establish the mechanical models of permeability coefficient, stress (strain) and damage, which is the core of the study of seepage-stress-damage coupling problem.

### 3.1. Percolation Characteristics of Mudstone Material

**Figure 1:**
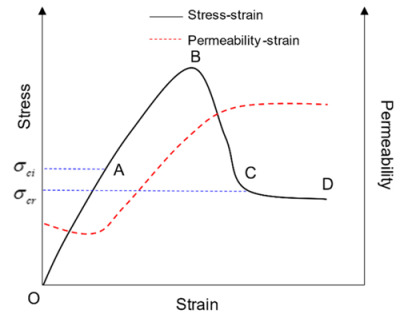
Typical permeability variation curve of mudstone.

According to the existing research results of permeability tests [24], the permeability test curve during the whole stress-strain process of mudstone shows segmental characteristics with the development of deformation. There are three characteristic sections: permeability reduction stage (OA section), permeability growth stage (AC section) and permeability stability stage (CD section). As shown in a typical mudstone permeability variation curve in Figure 1, there is a permeability mutation between OA and AC sections. This phenomenon can be explained by percolation theory.

Mudstone porous media consists of a solid framework and pores. For fluids, the solid framework is viewed as a “disconnection” bond, while pores are viewed as “connection” bonds. Randomly distributed pores are interconnected to form many pore groups called clusters or groups, of which, the group containing the most pores are called the largest group. In the initial stage (OA section) of mudstone deformation, the original pores are closed tightly, the porosity is decreased, the connectivity between pores in the mudstone is reduced, groups are in sporadic distribution, and some groups also have local connectivity but no infinite interpenetrating group is formed, leading to a decline in permeability. With the increase of axial stress, micro-cracks appear in the mudstone, pores are interconnected, and porosity increases. When the porosity increases to the critical value, the various groups within the mudstone interpenetrate to form an infinite group. The fluid can infiltrate the mudstone porous media completely along the percolation channel formed by the infinite group. At this time, mudstone permeability changes suddenly. This transformation is called percolation transformation. It can be seen that permeability evolution has obvious percolation characteristics (infiltration mutation) during the deformation and damage of mudstone. This paper establishes a permeability evolution equation based on percolation theory.

The study on percolation characteristics of mudstone shows that the analysis of pores (cracks) in mudstone is the basis for studying mudstone permeability [24]. Only when the size, distribution and connectivity of pores (cracks) in mudstone are clear, can we make a correct calculation and reasonable evaluation of permeability. Therefore, when studying mudstone permeability, size, distribution and connectivity of pores (cracks) can be determined according to the statistical rules of pores (cracks). However, this method has such shortcomings as uneasy operation, lack of regularity and disconnection between model parameters and actual parameters [13]. In this paper, based on the percolation theory, the variables that characterize micro-structure, such as size and distribution of pores (cracks) and their connectivity, are linked with macroscopic mechanical parameters (stress or strain) by connection probability, to establish the permeability evolution equation that can reflect changes of mudstone micro-structure during the damage.

### 3.2. Permeability Evolution Model

In this paper, the bond percolation model is adopted, the connection probability of any bond in the mudstone percolation model grid is p. The two bonds are interconnected and only the adjacent bonds are connection bonds. The pores (cracks) inside the mudstone are randomly distributed, under the action of external force, the dynamic evolution of pores (cracks) is related to the destruction of the micro-units in the rock. It can be known from the physical meaning of connection probability and destruction probability that connection probability p can be expressed as micro-unit destruction probability.

The damage of rock micro-units is random, and the distribution intensity function of micro rock destruction probability is in regard to strength of micro-units is ρ(F). Assuming that the micro-units follow Weibull distribution, the distribution intensity function is:

(17) $$\rho (F)=\frac{\gamma }{F_{0}}{\left(\frac{F}{F_{0}}\right)}^{\gamma -1}\exp{\left[-\left(\frac{F}{F_{0}}\right)\right]}^{\gamma }$$

Then, connection probability p can be obtained from Equation (17):

(18) $$p=\int_{0}^{F}\rho \left(x\right)dx=1-\exp\left[-{\left(F/F_{0}\right)}^{\gamma }\right]$$

where, F is the rock micro unit strength; $F_{0}$ and γ are Weibull distribution parameters, reflecting the mechanical properties of rock materials. Studies have shown that micro unit strength of rock can be represented by the axial strain of rock [25]. Then, Equation (18) can be expressed as:

(19) $$p=1-\exp{\left[-\left(\varepsilon_{1}/\varepsilon_{0}\right)\right]}^{\gamma }$$

where, $\varepsilon_{1}$ is axial strain of the rock, $\varepsilon_{0}$ is Weibull distribution parameter.

Parameter $\varepsilon_{0}$ can indicate the overall effect of micro unit strength on macroscopic damage. Parameter $\varepsilon_{ci}$ is the strain at dilatancy point C, parameter γ represents heterogeneity degree of distribution, which also indicates brittle plasticity of the rock material. A small γ means a high rock plasticity. In this work, $\varepsilon_{0}$ is set as equal as $\varepsilon_{ci}$ and γ=1. Thus, the connection probability p can be written according to Equation (19):

(20) $$p=1-\exp\left[-\left(\varepsilon_{1}/\varepsilon_{ci}\right)\right]$$

As the connection probability p increases (or decreases) to $p_{ci}$, an infinite connectivity group-the percolation group, can be formed where some macroscopic nature of the system undergoes a qualitative change (such as circulation or clogging of fluid in the porous medium), and percolation phenomenon occurs. At dilatancy point C, value of $p_{ci}$ is called percolation threshold, which can be expressed as:

(21) $$p_{ci}=1-\exp\left[-\left(\varepsilon_{ci}/\varepsilon_{ci}\right)\right]=1-e^{-1}=0.63$$

Mudstone permeability is related to the degree of interconnectivity between internal fractures. The quantitative index reflecting the connectivity degree of fracture network is connectivity C. From Equations (20) and (21), in concrete terms, it is stipulated that when each fracture in the fracture network extends indefinitely (that is, both ends of the fracture are tangential to the boundary of the study area), that is, when an independent passageway can be formed, its connectivity is equal to 1; on the contrary, if each group of cracks in the network does not intersect each other and no passageway is formed, the connectivity is equal to zero.

Connectivity C can be obtained by directly measuring the average number of intersections where each fracture in the fracture network intersects with other fractures. Generally, connectivity C can be expressed as a function of the number of fractures in the infinite group throughout the entire fracture network,

(22) $$C\propto {\left(N-N_{ci}\right)}^{2}$$

In Equation (22), N is the total number of fractures, and $N_{ci}$ is the critical number of fractures.

Through the above analysis, it can be seen that $\left(N-N_{ci}\right)$ is identical with $\left(p-p_{ci}\right)$ from Equation (22). Therefore, connectivity in a random micro-fracture network can be expressed as:

(23) $$C\propto {\left(p-p_{ci}\right)}^{2}$$

At some point, connectivity is the ratio of permeability at this point of time to the final permeability (permeability at full connectivity) $k_{s}$:

(24) $$C\propto k/k_{s}$$

By combining the Equations (22)–(24), permeability can be expressed as following with help of constant *A*:

(25) $$k=Ak_{s}{\left[\left(p-p_{ci}\right)\right]}^{2}=Ak_{s}{\left[\left(1-\exp\left(-\varepsilon_{1}/\varepsilon_{ci}\right)-p_{ci}\right)\right]}^{2}$$

When permeation reaches a peak, $k=k_{s}$, constant A can be determined from Equation (26).

(26) $$A={1/\left[1-\exp\left(-\varepsilon_{cc}/\varepsilon_{ci}\right)-p_{ci}\right]}^{2}$$

where $\varepsilon_{cc}$ is the axial strain at the permeation peak value.

According to the percolation theory, after the percolation transformation, the rock changes from impermeable to permeable. The above Equation (25) only shows the permeability expression after the percolation transformation, but cannot describe the evolution of rock permeability before the dilatancy point, so it is necessary to study the permeability evolution equation before percolation (before dilatancy).

(1) Permeability evolution equation before dilatancy (without damage)

Based on the Kozeny–Carman equation, the relationship between permeability coefficient k and volume strain $\varepsilon_{v}$ can be derived [26,27]:

(27) $$k=k_{0}\frac{1}{1+\varepsilon_{v}}{\left(1+\frac{\varepsilon_{v}}{n_{0}}\right)}^{3}$$

where, $k_{0}$ is initial permeability coefficient; $\varepsilon_{v}$ is volume strain; $n_{0}$ is initial porosity.

(2) Permeability evolution equation after dilatancy (damage zone)

It can be seen from Equation (25) that the rock permeability coefficient at dilatancy point C is zero, which is inconsistent with the actual situation. In fact, the permeability coefficient at the dilatancy point is:

(28) $$k_{c}=k_{0}\frac{1}{1+\varepsilon_{ci}^{v}}{\left(1+\frac{\varepsilon_{ci}^{v}}{n_{0}}\right)}^{3}$$

where, $\varepsilon_{ci}^{v}$ is the volume strain at dilatancy point C.

According to Equation (27) and Equation (28), the modification of Equation (25) is as follows:

(29) $$k=Ak_{s}{\left[\left(1-\exp\left(-\varepsilon_{1}/\varepsilon_{ci}\right)-p_{ci}\right)\right]}^{2}+k_{c}$$

From Equation (29), permeability evaluation during rock excavation can be fully described.

## 4. Numerical Simulation and Application

Based on the established seepage-stress damage coupling model of mudstone, the evolution process and permeability evolution of the damaged area during excavation of a mudstone roadway are studied.

To embed the proposed stress-seepage damage coupling model into the numerical model, the large-scale the commercial finite element code-ABAQUS was used as the platform for secondary development. First, the subroutine, named GETVRM, was used to obtain the data of integral points. Then, three new codes were developed to calculation relationships between deformation, damage, and permeability. The relationship between deformation parameters and damage is used by the command “*Elastic, DEPENDENCIES = 1”. The relationship between strength parameters of mudstone and plastic strain is defined by the command “*Mohr Coulomb Hardening DEPENDENCIES = 1”. The relationship between permeability and strain is defined by code “*permeability, specific = 10,000, DEPENDENCIES = 1”. Finally, the subroutine “USDFLD. for” was run repeatedly to calculate and obtain the deformation, damage, and permeability. 

### 4.1. Numerical Model Validation

In order to verify the effectiveness of the numerical model, the conventional triaxial compression test was conducted by finite element analysis and an experimental test at the same time. The model parameters can be obtained from Equation (30).

(30) $$D=1-\exp\left(0.013/\varepsilon_{v}+4.43\right)$$

**Figure 2:**
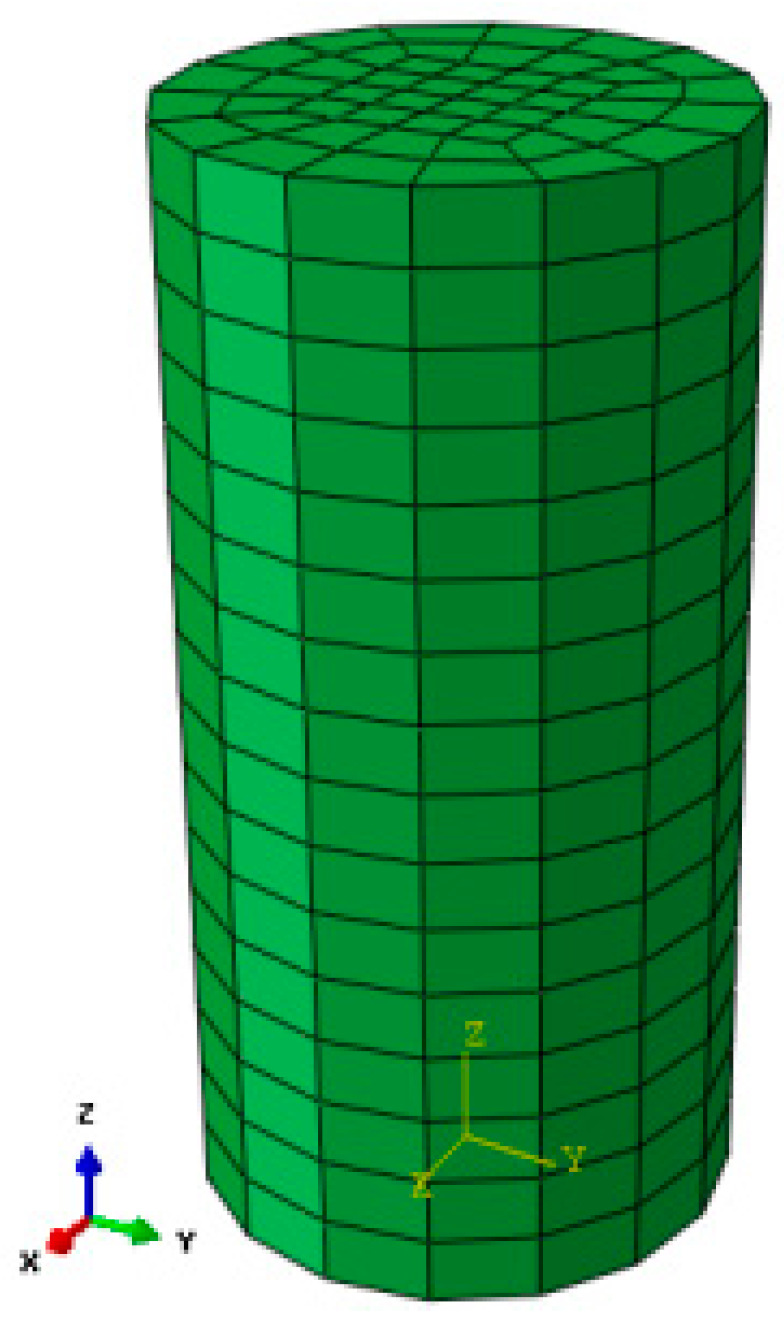
Triaxial test finite element calculation model.

The model size is a cylinder with the diameter of 50 mm and a height of 100 mm (as shown in Figure 2), and the influence of gravity is not considered in the simulation. Two different confining pressures, 0.89 MPa and 2.85 MPa, were applied during the test. The triaxial compression test curves of mudstone in the literature [28], and the established elastoplastic damage constitutive model of mudstone is used to fit the stress-strain curve of the mudstone. As shown in Figure 2, the numerical model is three-dimensional and has 720 hexahedral 8-node finite elements. 

**Figure 3:**
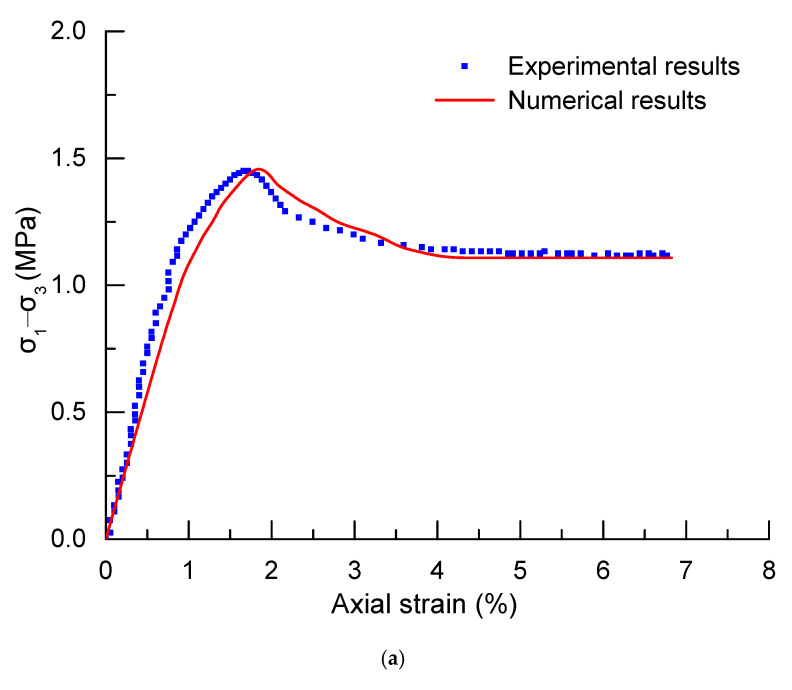

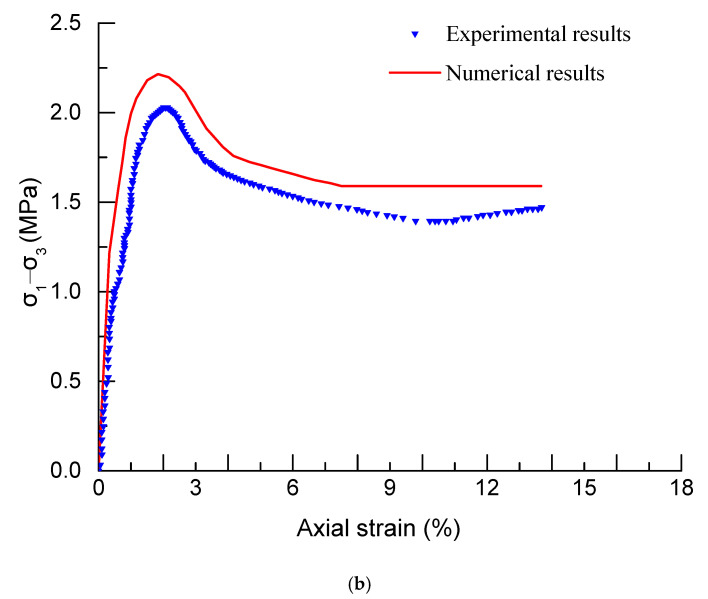
Comparisons between experimental and numerical values. (**a**) Confining pressure is 0.89 MPa. (**b**) Confining pressure is 2.85 MPa.

The comparisons between experimental and numerical results are shown in Figure 3. It can be seen from Figure 3 that the simulation results are consistent with the experimental results, which validates the numerical models and indicates that the elastoplastic damage constitutive model in this paper can reasonably describe the characteristics of the principal mechanical behaviour and the deformation characteristics. 

As shown in Figure 3, the maximum deviation between experimental and numerical results are about 8.63%, and 10.24% for corresponding confining pressure of 0.89 MPa, and 2.85 MPa, respectively. The results show a relatively high error, which may be caused by the following reasons.

Firstly, there are some differences between numerical simulation and the real laboratory test for triaxial compression test. For the laboratory test, there is a space between the pressing plate and the specimen. Besides, the confining pressure generated by the oil pressure applied to the rubber sleeve will produce a shear stress on the specimen. Both of these two reasons make the experimental results lower than the numerical results. 

Secondly, as is widely known, rock is one of the most typical heterogeneous materials, which causes the data deviation of rock materials to be relatively large. As a result, the mechanical parameters of rock materials are different from each other, which is the main reason of deviation between experimental and numerical results. 

Finally, the numerical results agree well with the target values obtained from laboratory tests. The results are acceptable for rock materials with all errors controlled to 20%.

### 4.2. Application of Proposed Model

**Figure 4:**
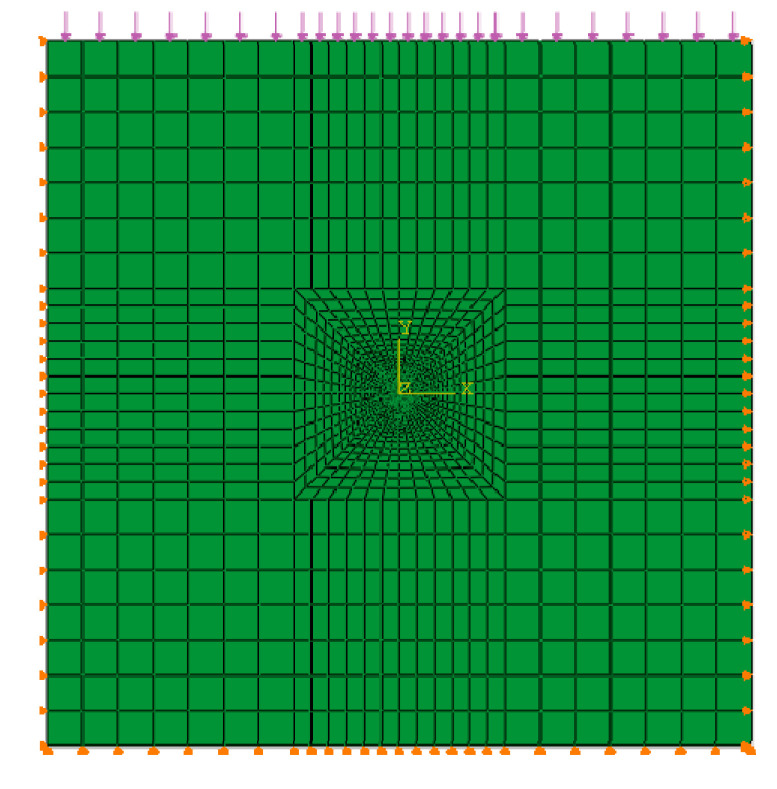
Finite element numerical model.

A roadway is located at a buried depth of 200 m, with a diameter of 6 m. The calculation model is shown in Figure 4. The model has a size of 100 m × 100 m with an excavation diameter of 6 m. The model contains 676 finite elements and these elements are a 4-node rectangle. The Y-direction displacement constraint is applied to the bottom while the X-direction displacement constraint is applied to the left and right sides of the model. The pressure stress is applied at the top of the model with a value of 3.5 MPa. The initial effective vertical stress at the top of the model is 1.75 MPa, the lateral pressure coefficient is 0.9 and the initial pore water pressure is 1.75 MPa. The mudstone has very low permeability. 

In the numerical simulation, it is assumed that the boundary is impermeable during excavation, and water permeability of the lining is considered after the completion of construction. The permeability coefficient evolution equation uses the expression given in Section 3.2, where the parameter A=50, and permeability coefficient k at mudstone rupture increases by three orders of magnitude compared with the original rock. The mudstone and lining material parameters are listed in Table 1. Other model parameters are listed in Equation (31).

(31) $$\left\{ \begin{array}{l} D=1-\exp\left(0.013/\varepsilon_{v}+4.43\right)\\ \sigma_{ci}=0.13\sigma_{3}+1.05\\ \sigma_{cc}=0.35\sigma_{3}+1.27\\ c\left({\bar{\varepsilon }}_{p}\right)=c_{0}+208.33\left(c_{r}-c_{0}\right){\bar{\varepsilon }}_{p} \end{array}\right.$$

The above calculation model is numerically calculated to study the spatial and temporal evolution of pore pressure, damage and the permeability coefficient during the roadway construction. The calculation results are as follows:

(1) Pore pressure

**Figure 5:**
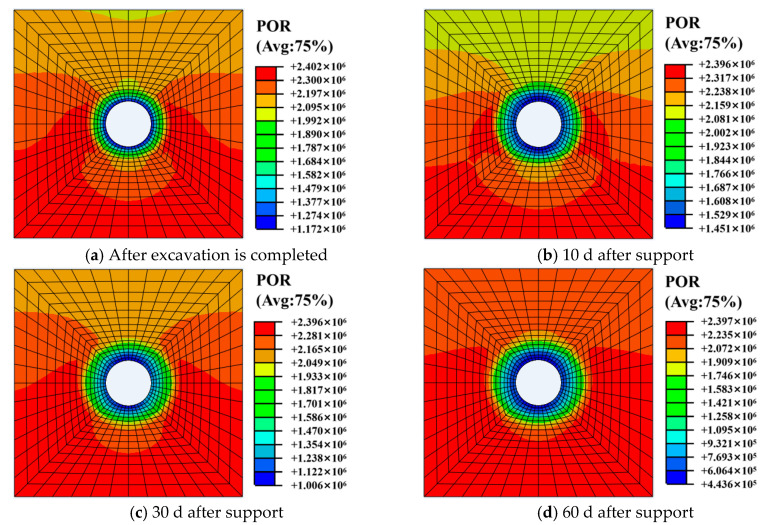
Pore pressure distribution of surrounding rock at different times.

**Figure 6:**
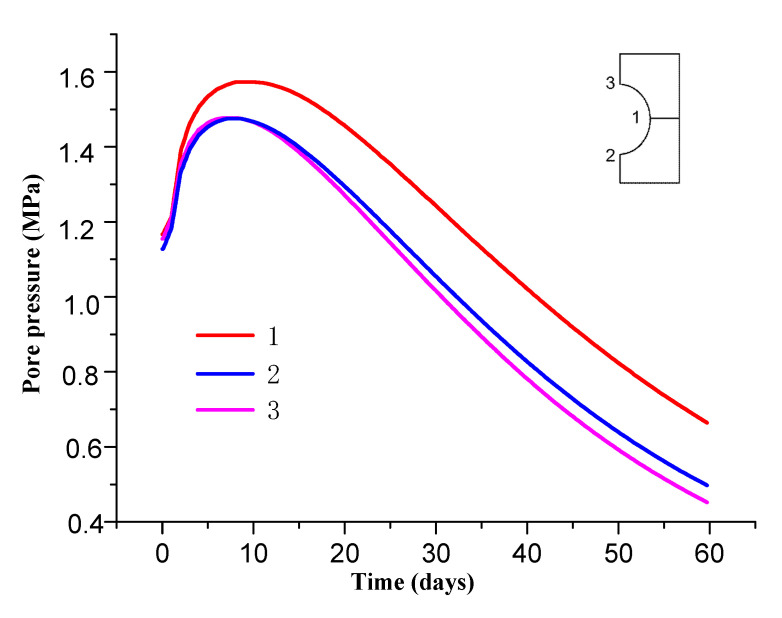
Variation curve of pore pressure of surrounding rock over time.

Figure 5 shows the distribution of pore pressure of surrounding rock at different times. Figure 6 shows the variation curve of pore pressure of surrounding rock over time. It can be seen from Figure 5 that pore water pressure suddenly drops to about 1.2 MPa after roadway excavation. Pore pressure gradually increases with the increase of time and reaches the maximum value of 1.4–1.6 MPa after about 12 d of roadway support. Afterwards, pore pressure gradually decreases and pore pressure of the inner wall of the surrounding rock decreases to 0.45–0.65 MPa after 60 d of support. 

**Figure 7:**
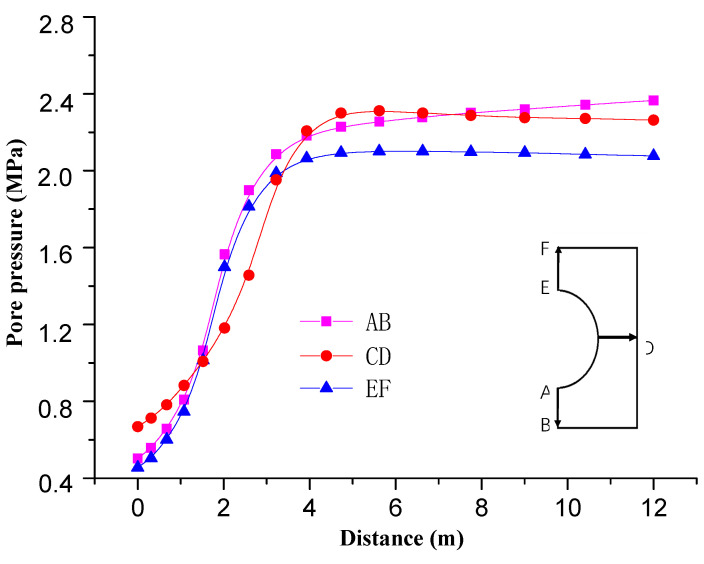
Variation curve of pore pressure at different positions of roadway.

Figure 7 shows the pore pressure changes along the roadway at different locations. It can be seen from Figure 6 that the range of influence of pore pressure on the sides of roadway is greater than that on the roof and floor due to uneven ground stress after roadway excavation. The former is about 4 m, while the latter is about 3 m.

(2) Damage Zone Development

**Figure 8:**
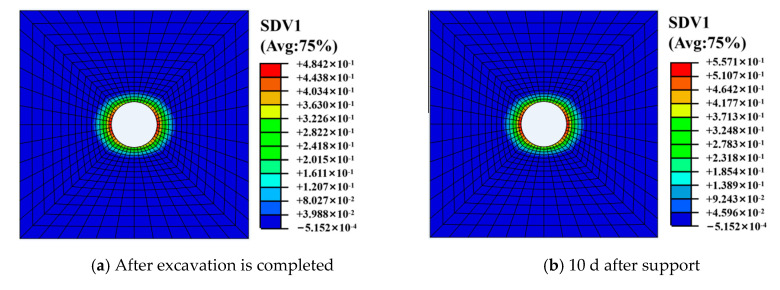

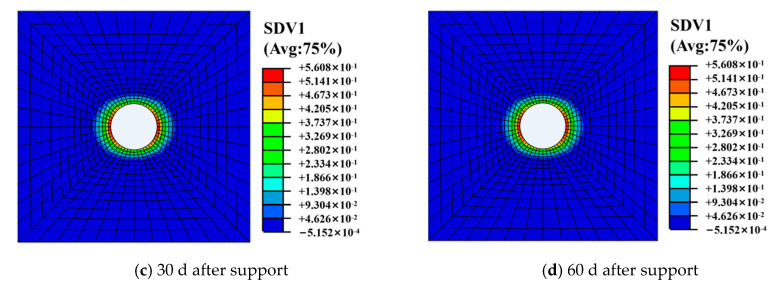
Distribution of surrounding rock damage at different times.

**Figure 9:**
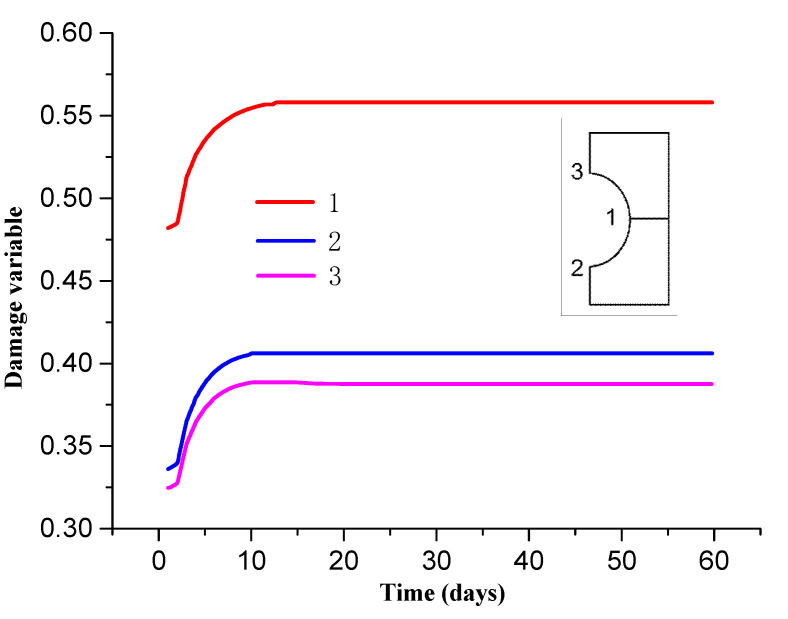
Distribution curve of surrounding rock damage over time.

Figure 8 and Figure 9 show the distribution of the surrounding rock damage zone at different times. It can be seen from the Figure 9 that the surrounding rock is damaged after the roadway excavation. The damage value is 0.3–0.5. The damage degree of the surrounding rock gradually increases with the increase of time, and reaches the maximum of 0.38–0.56 after about 10 d of roadway support. The damage degree tends to be stable thereafter. 

**Figure 10:**
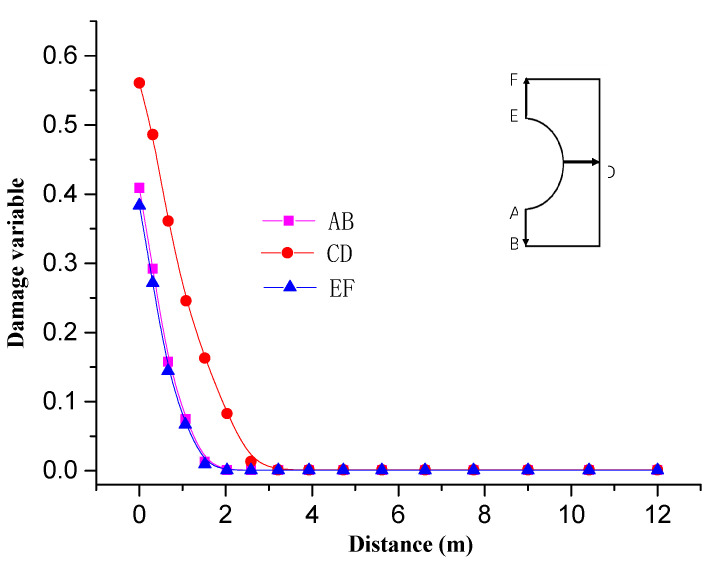
Distribution of surrounding rock damage at different locations in the roadway.

Figure 10 shows the damage area distribution of surrounding rock along the roadway at different locations. It can be seen from Figure 8 that the range of damage on the sides of roadway is larger than that on the roof and floor due to difference stress between horizontal and vertical directions. The damage zone range of the former is about 3 m, while the latter is about 2 m.

(3) Permeability coefficient

**Figure 11:**
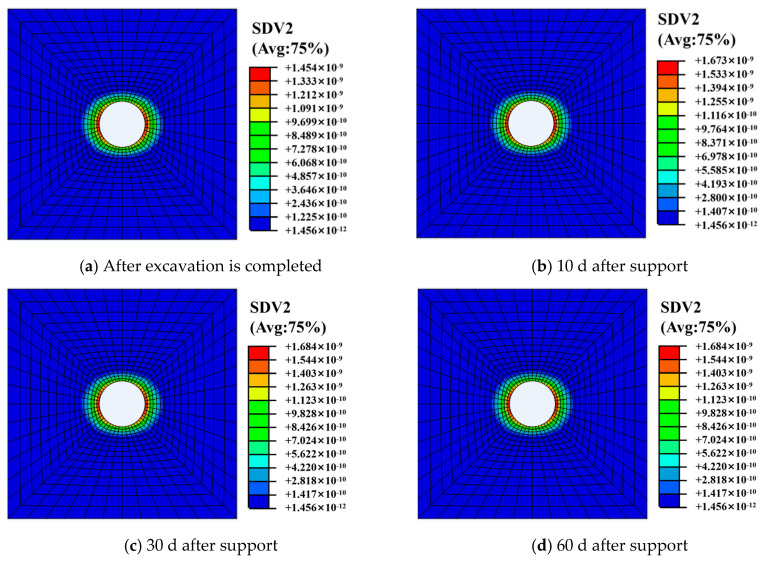
Distribution of rock permeability coefficient.

**Figure 12:**
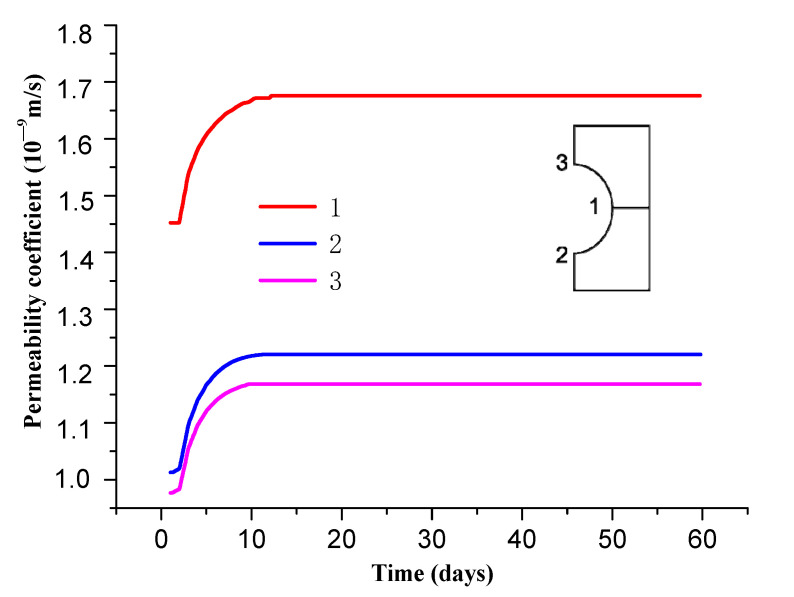
Variation curve of permeability coefficient of surrounding rock over time.

Figure 11 and Figure 12 show the distribution of the surrounding rock permeability coefficient over time. It can be seen from the Figure 12 that permeability coefficient of surrounding rock is obviously increased after the tunnel excavation, varying in the range of 0.9 × 10^−9^–1.5 × 10^−9^ m/s. The permeability of surrounding rock gradually increases with the increase of time, and reaches its maximum of 1.15 × 10^−9^–1.7 × 10^−9^ m/s after about 10 d of roadway support, which is 400~600 times that of the original rock permeability coefficient. The permeability coefficient tends to be stable thereafter. 

**Figure 13:**
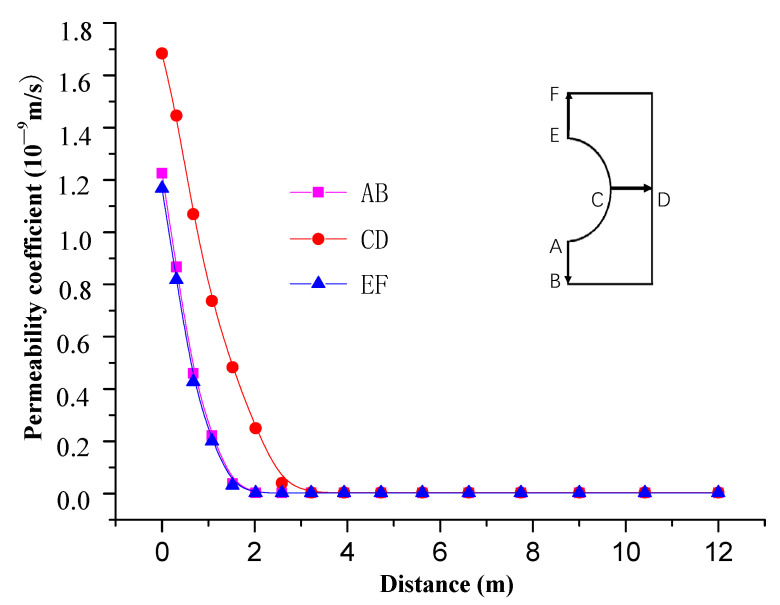
Distribution of permeability coefficient at different locations of roadway.

Figure 13 shows the distribution of the surrounding rock permeability coefficient along the roadway at different locations. It can be seen from Figure 11 and Figure 13 that permeability of on the sides of roadway is larger than that on the roof and floor due to difference stress. The former is about 3 m, while the latter is about 2 m.

## 5. Conclusions

In this paper, the permeability evolution before and after capacity expansion in the process of total stress-strain is analyzed. On basis of the results, first, an elastoplastic damage constitutive equation and a permeability evolution model, which can consider the damage and permeability mutation of mudstone, was proposed. Then, the proposed model was embedded into a numerical method and validated. The main conclusions can be drawn as follows:

(1) Permeability variation curves includes three stages, namely permeability reduction, permeability growth and permeability stability, during the deformation process of mudstone. Furthermore, there is a sudden permeability change after rock capacity expansion, which is called the percolation characteristic of mudstone.
(2) The percolation characteristics of mudstone is mainly caused by propagation and forming an infinite group of micro-cracks during the loading process.
(3) Based on percolation theory, the permeability evolution equation describing the micro-macro hydraulic behavior of mudstone is deduced. Furthermore, a damage variable is introduced into the proposed model based on the principle of minimum energy consumption and seepage-stress damage coupling model of mudstone is established.
(4) The proposed stress-seepage damage coupling model was embedded into commercial finite element code-ABAQUS, and the numerical codes were validated by comparing experimental and numerical results.
(5) The proposed theoretical and numerical modes were applied on a roadway construction process. The results show that over time pore pressure first increases and then decreases, while damage and the permeability coefficient gradually increase and finally nearly remain constant.

## Figures and Tables

**Table 1:** Material parameters of finite element model.

	Weight (kN/m^3^)	Elasticity Modulus (GPa)	Poisson’s Ratio	Cohesion (MPa)	Residual Cohesion (MPa)	Internal Friction Angle (°)	Permeability Coefficient (m/s)	Porosity
Mudstone	20	0.6	0.13	0.3	0.06	19	3 × 10^−12^	0.39
Lining	25	20	0.25	-	-	-	1.2 × 10^−11^	0.06
